# Dependence of the electrical and optical properties on growth interruption in AlAs/In_0.53_Ga_0.47_As/InAs resonant tunneling diodes

**DOI:** 10.1186/1556-276X-6-603

**Published:** 2011-11-23

**Authors:** Yang Zhang, Min Guan, Xingfang Liu, Yiping Zeng

**Affiliations:** 1 Key Laboratory of Semiconductor Materials Science, Chinese Academy of Sciences, P. O. Box 912, Beijing, 100083, People's Republic of China; 2 Material Science Center, Institute of Semiconductors, Chinese Academy of Science, P. O. Box 912, Beijing 100083, People's Republic of China

**Keywords:** resonant tunneling diode, *I-V *characteristics, molecular beam epitaxy

## Abstract

The dependence of interface roughness of pseudomorphic AlAs/In_0.53_Ga_0.47_As/InAs resonant tunneling diodes [RTDs] grown by molecular beam epitaxy on interruption time was studied by current-voltage [*I-V*] characteristics, photoluminescence [PL] spectroscopy, and transmission electron microscopy [TEM]. We have observed that a splitting in the quantum-well PL due to island formation in the quantum well is sensitive to growth interruption at the AlAs/In_0.53_Ga_0.47_As interfaces. TEM images also show flatter interfaces with a few islands which only occur by applying an optimum value of interruption time. The symmetry of *I-V *characteristics of RTDs with PL and TEM results is consistent because tunneling current is highly dependent on barrier thickness and interface roughness.

## Introduction

The terahertz [THz] frequency range is receiving considerable attention recently due to various applications. Resonant tunneling diodes [RTDs] have been considered as one of the promising candidates [1-3] for the compact and coherent solid-state THz source at room temperature. Similar to all other quantum-effect devices, one of the most challenging tasks in fabricating RTDs is the control of interface roughness in the quantum-well region. Interface roughness has often been discussed as a factor in determining current through RTDs. Monolayer [ML] fluctuations in tunnel barrier thickness are expected to be important because it is necessary to use tunnel barriers only a few ML thick to obtain the high-peak current densities required for high-speed devices. There have been many theoretical discussions on the importance of surface roughness and interface scattering mechanisms that contribute to peak and valley currents in (In, Ga)As/AlGaAs RTDs and AlSb/InAs RITDs [4-6]. On the experimental sides, there have been reports aimed at linking barrier roughness measured by scanning tunneling microscopy or photoluminescence [PL] spectrum [7,8]. Therefore, a deeper understanding of the structural imperfections in RTDs grown by molecular beam epitaxy [MBE] and their effects on the electrical characteristics of RTDs is needed in order to reproducibly obtain such RTDs.

In this paper, we report the interface roughness and electrical characteristics of pseudomorphic AlAs/In_0.53_Ga_0.47_As/InAs RTDs' dependence on growth interruption. Though the electrical properties showed anomalous growth interruption dependence, we found an optimum growth condition for increasing the current-voltage [*I-V*] characteristics' symmetry of RTDs. We also examined the relation between electrical properties and structural imperfections in RTDs. The competition relationship in a PL-integrated intensity of excitonic transitions reveals that the PL spectrum is sensitive to quantum-well-width fluctuation. In addition, transmission electron microscopy [TEM] images also show flatter AlAs/In_0.53_Ga_0.47_As interfaces with a few islands which only occur by applying an optimum value of interruption time.

### Experiment

**Figure 1:**
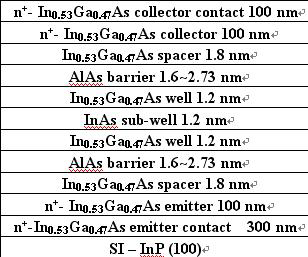
**Schematic cross section of an AlAs/In_0.53_Ga_0.47_As/InAs RTD**.

The epitaxial structure of the AlAs/In_0.53_Ga_0.47_As/InAs RTDs was grown by the solid-source MBE system (Veeco, New York, NY, USA) on a (100)-oriented semi-insulating, 3-inch InP substrate. The layer structure from the top of the RTDs is as follows: cap n^+^-In_0.53_Ga_0.47_As/collector n^+^-In_0.53_Ga_0.47_As/spacer In_0.53_Ga_0.47_As/barrier AlAs/well In_0.53_Ga_0.47_As/sub-well InAs/well In_0.53_Ga_0.47_As/barrier AlAs/spacer In_0.53_Ga_0.47_As/emitter n^+^-In_0.53_Ga_0.47_As/buffer n^+^-In_0.53_Ga_0.47_As. Figure 1 shows the schematic cross section of an RTD. An InAs sub-well thinner than the estimated critical thickness was inserted between the middle of the In_0.53_Ga_0.47_As well layer to lower the well energy level. The substrate temperature during the growth of the In_0.53_Ga_0.47_As layers and AlAs barrier layers was maintained at 480°C. However, the substrate temperature was lowered to 420°C when the InAs sub-well layer was grown. This growth temperature of sub-well InAs is appropriate for InAs grown on the In_0.53_Ga_0.47_As well. By adjusting growth interruptions of 10, 25, 40, 50, and 60 s at the In_0.53_Ga_0.47_As/InAs interfaces, we could investigate the dependence of interface roughness on interruption time; in order to simplify the influence of other growth interruptions on In_0.53_Ga_0.47_As/InAs interfaces, all samples on the interface of In_0.53_Ga_0.47_As/InAs adopted the same interruption time of 40 s.

**Figure 2:**
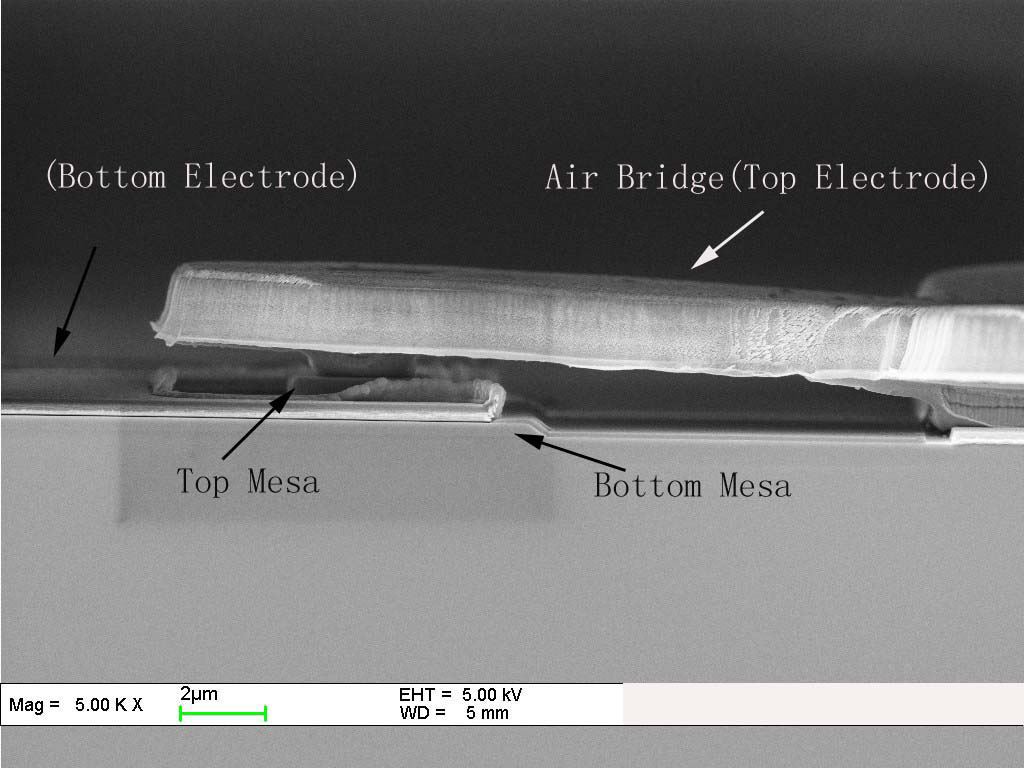
**SEM photograph of a fabricated AlAs/In_0.53_Ga_0.47_As/InAs RTD-based InP substrate**.

RTD devices with contact areas (collector) ranging from 2 × 2 μm^2 ^to 10 × 10 μm^2^, defined using electron beam and conventional UV-line lithography techniques, were fabricated by wet-etching and lift-off processes in a microwave-compatible, two-step mesa technology. Devices were covered with Si_3_N_4 _as a passivation layer. Ohmic contacts were made using Ti/Pt/Au for the top and bottom contacts. In that case, the devices were connected to low-loss transmission lines by means of air-bridge interconnection which largely reduced parasitic capacitance. A scanning electron microscopy [SEM] of a representative device is shown in Figure 2. As clearly shown, one 2-μm-thick air bridge interconnects the top mesa and the pad. The DC *I-V *characteristics of RTDs were measured in the negative differential resistance region with a KEITHLEY4200 semiconductor parameter analyzer (KEITHLEY, Cleveland, OH, USA) at room temperature. The PL measurements were performed at 77 K with an Ar-ion (514.5 nm) laser as the excitation source and a liquid nitrogen-cooled InSb detector for receiving the PL signal. TEM observations were carried out using a JEM-2010 (JEOL, Tokyo, Japan) operated at 200 KV with a resolution of 0.19 nm.

## Results and discussion

**Figure 3:**
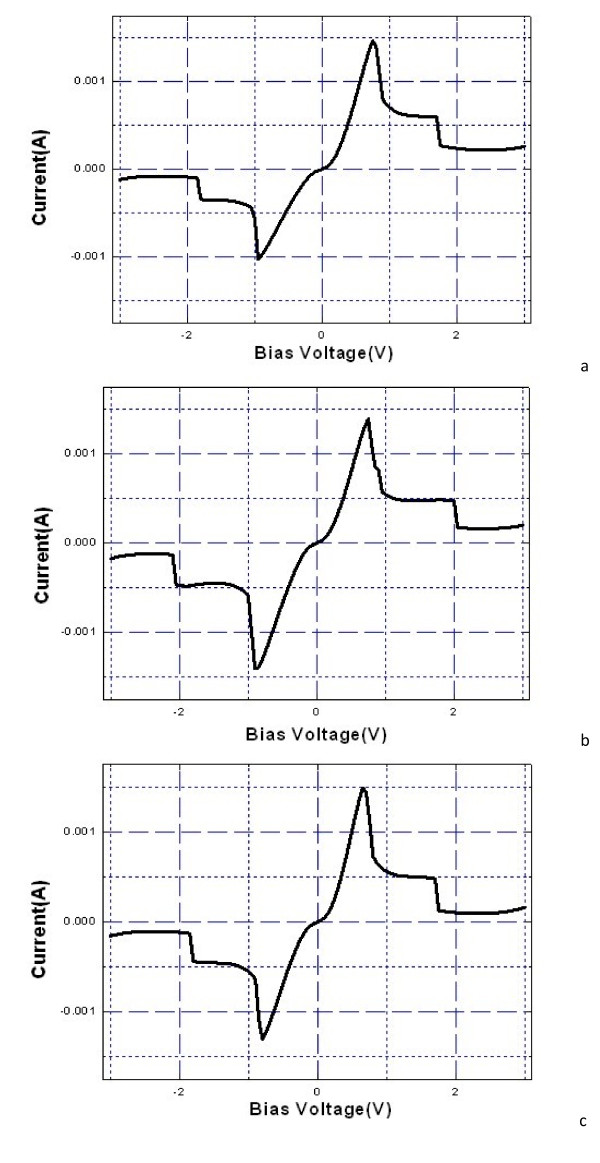
***I-V *characteristics of RTDs with growth interruptions**. (**a**) 10, (**b**) 40, and (**c**) 60 s.

Figure 3 shows the typical *I-V *characteristics of RTDs of 6 × 6-μm^2 ^contact areas with growth interruptions of 10, 40, and 60 s at the AlAs/In_0.53_Ga_0.47_As interfaces with an AlAs barrier thickness of 2.73 nm. Here, a positive bias means that the top of the mesa was biased positively with respect to the substrate, and a negative bias is the reverse. Figure 3b shows the symmetric *I-V *characteristics with a peak current density of 9.8 KA/cm^2 ^and a peak-to-valley ratio of 16. However, as the growth interruption decreases, the *I-V *characteristics become asymmetric, where *I*_p _in the negative bias decreases. In Figure 3a, I_p _in the positive bias is 1.5 times larger than that in the negative bias. Figure 3c shows the little asymmetric *I-V *characteristics, where *I*_p _in the positive bias increases and *I*_p _in the negative bias decreases relative to those in Figure 3b.

**Figure 4:**
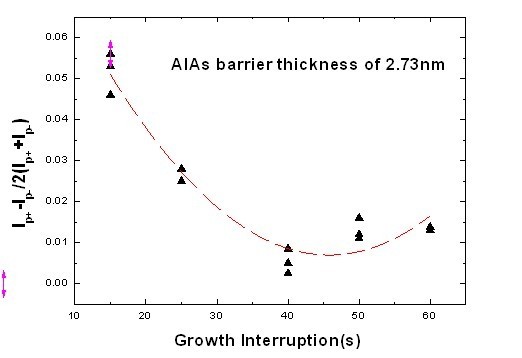
***I-V *asymmetry dependence on growth interruption**. *I-V *asymmetry dependence on growth interruption at the AlAs/In_0.53_Ga_0.47_As interfaces with an AlAs barrier thickness of 2.73 nm.

The dependence of *I-V *asymmetry on growth interruption with an AlAs barrier thickness of 2.73 nm and 6 × 6-μm^2 ^contact areas is shown in Figure 4, in which the degree of asymmetry was defined as *(I*_p+ _- *I*_p-_*)/2(I*_p+ _***+ ****I*_p-_), where *I*_p+ _and *I*_p- _are the peak currents in the positive and negative biases, respectively. As we can see in Figure 4, the degree of asymmetry decreases quickly with the increment of interruption time. However, when the interruption time exceeds 40 s at the AlAs/In_0.53_Ga_0.47_As interface, the degree of asymmetry rises again. As shown in Figure 4, *I-V *asymmetry is very sensitive to interruption time. Therefore, to improve *I-V *symmetry, the interruption time at the AlAs/In_0.53_Ga_0.47_As interfaces should be enhanced by at least 30 s. An interruption time longer than 40 s should be adopted according to our experiment.

**Figure 5:**
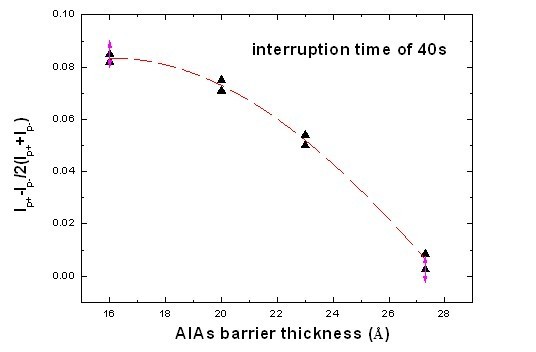
***I-V *asymmetry dependence with an AlAs barrier thickness of 1.6 ~ 2.73 nm**. *I-V *asymmetry dependence with an AlAs barrier thickness of 1.6 ~ 2.73 nm and an interruption time of 40 s at the AlAs/In_0.53_Ga_0.47_As interfaces.

Figure 5 reveals *I-V *asymmetry dependence on an AlAs barrier thickness of 1.6 ~ 2.73 nm with an interruption time of 40 s at the AlAs/In_0.53_Ga_0.47_As interfaces. As we can see in Figure 5, with the increment of the AlAs barrier thickness, *I-V *asymmetry has been effectively dropped. The possible origins of the asymmetry in the *I-V *characteristics are mainly the fluctuation of the AlAs barrier around the well-structure layer and asymmetry in the interface roughness [9]. Although the barrier thickness changes from 1.6 to 2.73 nm, the tunneling current will cause changes in magnitude. However, the *I-V *characteristics' asymmetry with a thinner barrier is greater than that with a thicker barrier. Therefore, to improve *I-V *symmetry, the growth of RTD with a thinner barrier should be done by other methods to facilitate a smooth interface of the barrier, such as a migration-enhanced epitaxy.

**Figure 6:**
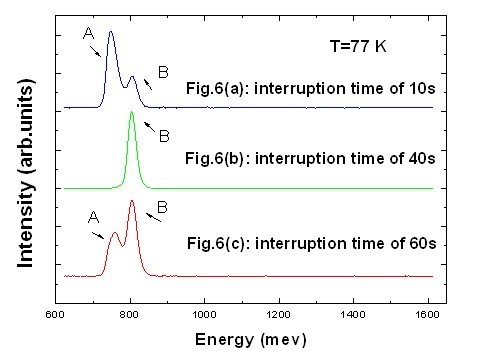
**PL spectra of RTDs with interruption times**. (**a**) 10, (**b**) 40, and (**c**) 60 s.

Figure 6 shows representative PL spectra from three of the samples with interruption times of 10, 40, and 60 s at the AlAs/In_0.53_Ga_0.47_As interfaces. Obviously, the existence of multiple PL peaks and the ratio of their integrated intensity [*I*_PL_] of luminescence are strongly dependent on interruption time at the AlAs/In_0.53_Ga_0.47_As interfaces. In Figure 6a, c, with interruption times of 10 and 60 s, respectively, the PL spectra are dominated by two luminescence peaks, in which low-energy peak A is at about 755 mev and peak B is at 805 mev, while there is only a luminescence peak B (804.7 mev) with an interruption time of 40 s in Figure 6b. All three cases show similar spectra in that there is a high-energy peak B at 805 mev which mainly originates from the first electron and heavy-hole radiation recombination in quantum well [QW], while the low-energy peak A is probably caused by interface roughness of QW in the atomic layer scale. It is important to note that the full width at half maximum [FWHM] value for an interruption time of 40 s is 18.4 mev which is the narrowest FWHM value in the three samples of the high-energy peak B at 77 K.

With respect to the study of PL spectra of different interruption times at the AlAs/In_0.53_Ga_0.47_As interfaces, we present the following explanation and discussion: (1) In contrast to high-energy peak B, the presence of peak A and the ratio of *I*_PL _(A) and *I*_PL _(B) are more sensitive to interruption time at the AlAs/In_0.53_Ga_0.47_As interfaces; however, the interruption time is closely related to interface roughness [10]. These phenomena could be explained by considering AlAs/In_0.53_Ga_0.47_As interface problems, such as well-size fluctuations in the atomic layer scale, rather than impurity effects since a low-energy peak is not observed in samples with an interruption time of 40 s. (2) As reported in a previous work [11], with increasing interruption time at interfaces, sufficient time allows cation to migrate and smooth the interfaces, whereas with increasing interruption time beyond 60 s, we found that peak A reemerges in the PL spectra. Therefore, we believe that interface smoothness of a long interruption is commonly better than that of a short interruption. However, this is not the best choice for strained AlAs/In_0.53_Ga_0.47_As/InAs RTDs. Then, there must be another significant contribution to the degradation of interface smoothness for a longer interruption time. Normally, after the InAs sub-well was grown, we increased the substrate temperature for the growth preparation of the top In_0.53_Ga_0.47_As well layer. The lattice mismatch between In_0.53_Ga_0.47_As and InAs is 3.1%, which is considerably large. So with a too long interruption time, which means an *in situ *anneal, a strain-induced roughness in a highly strained In_0.53_Ga_0.47_As layer might play an important role on interface smoothness. In addition, according to the observation of an RHEED pattern, when the interruption time exceeded 60 s, streak pattern of the In_0.53_Ga_0.47_As layer was weaker than before. Therefore, strain-induced roughness is probably viewed as the main cause of abnormal PL spectra.

**Figure 7:**
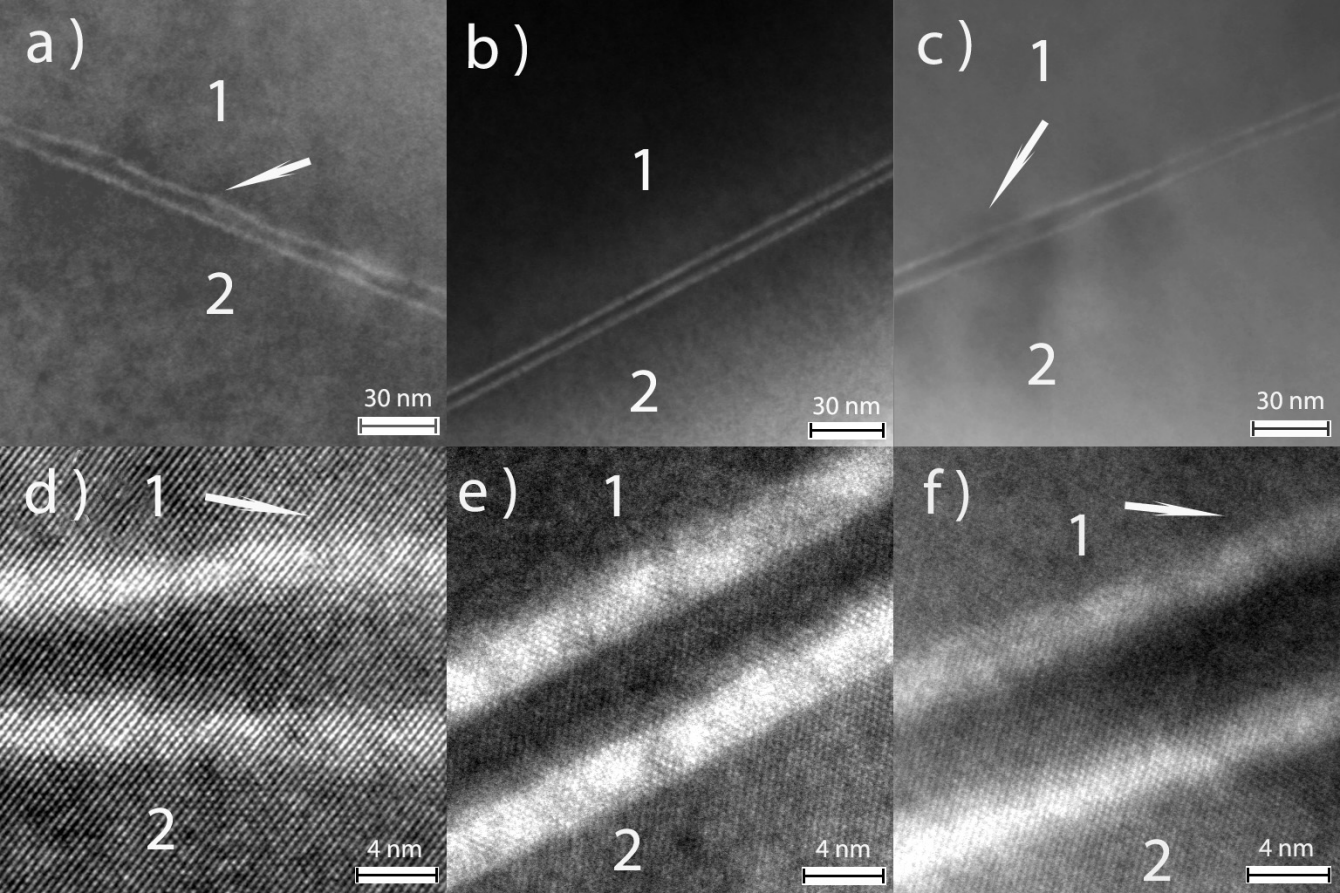
**TEM images of RTDs with growth interruptions**. (**a**) 10, (**b**) 40, (**c**) 60, (**d**) 10, (**e**) 40 and (**f**) 60 s.

Figure 7a, b, c shows TEM images of active regions of RTDs with interruption times of 10, 40, and 60 s at the AlAs/In_0.53_Ga_0.47_As interfaces. Figure 7d, e, f shows enlarged images of Figure 7a, b, c with a scale of 4 nm. In the images, the white lines are the AlAs barriers, where the upper lines correspond to the top barriers and the lower lines to the bottom barriers. '1' is the bottom In_0.53_Ga_0.47_As emitter layer, and '2' is the top In_0.53_Ga_0.47_As collector layer. The barriers with an interruption time of 40 s appear as two parallel lines, indicating the smooth interface of the AlAs barrier and In_0.53_Ga_0.47_As well. Figure 7a, d shows that the interface fluctuation of the bottom AlAs barrier further degenerates the interface roughness of the top In_0.53_Ga_0.47_As well and top AlAs barrier only because of an interruption time of 10 s at the AlAs/In_0.53_Ga_0.47_As interfaces. Figure 7c, f shows a little fluctuation of AlAs barriers and In_0.53_Ga_0.47_As well. As mentioned above, enough time allows cation to migrate freely along the interface for improving interface smoothness. However, a longer interruption time might favor a strain release that accommodates the mismatch between the InAs sub-well and In_0.53_Ga_0.47_As well. The detail of strain release is unclear at present. We need to further study the stress effects of RTD growth. TEM images show that there is a correspondence with PL spectra and *I-V *symmetry. In our study, the measurement of RTDs with different contact areas all show similar results.

## Conclusion

In this work, we have shown the dependence of interface roughness on interruption time by *I-V *characteristics, PL, and TEM. From the analysis of PL spectra and TEM, we have determined that there is an optimum value of interruption time at interfaces for the growth of highly strained AlAs/In_0.53_Ga_0.47_As/InAs RTDs rather than adopting too long interruption times. The results are very useful to further develop the applications of InP-based RTDs.

## Competing interests

The authors declare that they have no competing interests.

## Authors' contributions

YZ carried out the material growth by MBE. MG and XL carried out the device fabrication. YZ participated in the measurement of RTDs. All authors read and approved the final manuscript.
